# Predicting the Spatial Distribution and Severity of Soil Erosion in the Global Tropics using Satellite Remote Sensing

**DOI:** 10.3390/rs11151800

**Published:** 2019-07-31

**Authors:** Tor-Gunnar Vågen, Leigh Ann Winowiecki

**Affiliations:** World Agroforestry Centre (ICRAF), P.O. Box 30677-00100 Nairobi, Kenya

**Keywords:** soil erosion, land degradation, global tropics, food security, remote sensing, Earth observation, ecosystem health, resilience

## Abstract

This research paper presents the first consistent spatial assessments of soil erosion prevalence based on Earth observation data and systematic field surveys to train models that predict soil erosion prevalence across the global tropics. The results of the study have major implications for efforts to identify land degradation hotspots and design interventions to restore degraded lands given the importance of soil erosion as a major process of land degradation globally. The paper also presents analysis of changes in soil erosion prevalence over the 15 year period from 2002 to 2017. The results of the study can also be used to better understand ecosystem health and resilience in the global tropics, for assessments of food security, and the implications of land degradation on climate change.

Soil erosion has long been recognized as a major process of land degradation globally, affecting millions of hectares of land in the tropics and resulting in losses in productivity and biodiversity, decreased resilience of both marine and terrestrial ecosystems, and increased vulnerability to climate change. This paper presents an assessment of the extent of soil erosion in the global tropics at a moderate spatial resolution (500 m) based on a combination of systematic field surveys using the Land Degradation Surveillance Framework (LDSF) methodology and Earth observation data from the Moderate Resolution Imaging Spectroradiometer (MODIS) platform. The highest erosion prevalence was observed in wooded grassland, bushland, and shrubland systems in semi-arid areas, while the lowest occurrence was observed in forests. Observed erosion decreased with increasing fractional vegetation cover, but with high rates of erosion even at 50–60% fractional cover. These findings indicate that methods to assess soil erosion need to be able to detect erosion under relatively dense vegetation cover. Model performance was good for prediction of erosion based on MODIS, with high accuracy (~89% for detection) and high overall precision (AUC = 0.97). The spatial predictions from this study will allow for better targeting of interventions to restore degraded land and are also important for assessing the dynamics of land health indicators such as soil organic carbon. Given the importance of soil erosion for land degradation and that the methodology gives robust results that can be rapidly replicated at scale, we would argue that soil erosion should be included as a key indicator in international conventions such as the United Nations Convention to Combat Desertification.

## Introduction

Soil loss through erosion is widely recognized as one of the most important and widespread forms of land degradation globally [1–3] and was reported as being a major threat to soil productivity as early as in the 1930s [4]. While studies have assessed global soil erosivity [5], potential soil loss [6], and soil erosion potential [7], the spatial extent and severity of soil erosion has not yet been consistently quantified. Estimates of both spatial and temporal dynamics of soil erosion are needed to improve progress towards the implementation of measures to reduce soil erosion. Efforts to reduce soil erosion have been largely ineffective to date despite the widespread recognition that it is an increasing problem [8–11]. This is particularly true in developing countries where population growth, agricultural expansion and mining, coupled with scarcity of land [12] and adverse economic and/or political policies [13] have resulted in there being little if any progress towards reducing land degradation through erosion. Also, up-to-date and spatially explicit estimates of soil erosion prevalence are needed for more effective targeting and scaling of efforts to avoid future land degradation. The importance of soil erosion goes beyond its widely recognized effects on agricultural productivity, with studies showing that the potential impacts of erosion on the global carbon budget, and, hence, climate change, can be significant [14,15]. These factors point to the need for improved methods to spatially assess the prevalence of soil erosion at scales relevant to management interventions, including the tracking of the occurrence and severity of erosion in landscapes over time.

Human activities are currently having profound impacts on the environment with some studies suggesting that human-driven processes of land degradation are threatening global food security [16] by impacting on the adaptive capacity and resilience of ecosystems worldwide, reducing their ability to sustain productivity and to provide other critical ecosystem services. Continued conversion of natural ecosystems into agricultural land is also leading to loss of habitats and biodiversity [17,18], and often to further increases in land degradation and the loss of critical ecosystem functions for regulation of water and nutrient cycling. Land degradation can also lead to increased risks to human health through the emergence and spread of infectious diseases [19], for example in deforested areas or in areas where wetlands are degraded resulting in sharp increases in malaria risk [20].

Although attempts have been made to assess the extent of global land degradation, either through expert opinion such as the Global Assessment of Land Degradation (GLASOD) [21,22], by using proxies such as the Normalized Difference Vegetation Index (NDVI) [23] or rain-use efficiency (RUE) [24], or through the use of empirical models such as the Revised Universal Soil Loss Equation (RUSLE) [25], estimates remain crude at best. In some cases, such assessments (e.g., the use of NDVI as a proxy for land degradation) can potentially be misleading, resulting in the implementation of costly interventions that are not suitable for the restoration of degraded ecosystems. Reference [7] applied the RUSLE empirical model to predict global soil erosion using a combination of factors derived from: (1) the Shuttle Radar Topography Mission (SRTM) and ASTER GDEM digital elevation models (DEMs) for slope length and steepness (LS factor) and (2) the WorldClim database [26] for precipitation and temperature data to estimate erosivity (R factor), the MODIS Vegetation Continuous Fields product (VCF, MOD44B) as a proxy for vegetation (C factor), and World Soil Information (ISRIC) SoilGrids [27] for soil organic carbon to estimate soil erodibility (K factor). They produced a global map of the potential rates of soil erosion at 250 m spatial resolution, although the actual spatial resolution is likely to be lower due to the lower spatial resolution of some of the input parameters used such as WorldClim, which has a spatial resolution of 1 km by 1 km. Also, this study did not include validations against observed rates of soil erosion for the years modeled (2001 and 2012; [7]), which is challenging due to a general lack of field-based monitoring of soil erosion rates.

We present a study where we use Earth observation to detect and map the occurrence of soil erosion across the global tropics, defined here as the part of the Earth found within the boundaries of the parallels of 40^◦^ south and 40^◦^ north. The main objective of the study was to explore the application of Earth observation, which has been shown in the past to be promising at more local scales [28,29], to provide rapid assessments of soil erosion for spatially distributed monitoring. We combined systematically collected field observations of erosion with Earth observation data to predict the spatial and temporal distribution of soil erosion prevalence at moderate spatial resolution (500 m) using MODIS data for the years 2002, 2007, 2012, and 2017. The spatial assessments of erosion provide estimates of land degradation hotspots and can be combined with other indicators of ecosystem health, including social factors, to better assess and identify drivers of land degradation and target land management interventions to reverse degradation.

We also explored interactions between vegetation cover and soil erosion prevalence, as well as erosion prevalence by geographical regions and Holdridge life zones in the tropics [30]. While the mapping of Holdridge life zone regions globally remain coarse, they do present a useful framework within which processes of, for example, land degradation, soil condition, hydrological functioning, and climate change can be assessed, particularly in the context of ecological resilience and for understanding interactions between inherent properties of soils and landscapes and human influences. This study was part of wider efforts to collect critical datasets on ecosystem health that are consistent and can be applied to assess the state of ecosystems worldwide at multiple spatial scales, moving beyond simple descriptive analytics of land degradation processes such as soil erosion towards diagnostics and predictive analytics to help target interventions that avoid or reverse land degradation.

## Materials and Methods

This study used a combination of systematically sampled and characterized sites from across a range of different tropical ecosystems and remote sensing or Earth observation. As described in more detail below, field observations of soil erosion were used to train machine learning algorithms (models) to predict the occurrence of soil erosion based on moderate resolution satellite imagery over a period of 15 years from 2002 to 2017. In order to achieve consistent estimates of erosion, the timing (year) of field observations were matched with annual composites of satellite image reflectance data and the predictive models were tested using an independent dataset.

### Field Data Collection

**Figure 1:**
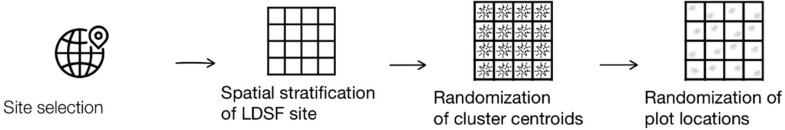
Schematic illustration of the Land Degradation Surveillance Framework (LDSF) sampling framework showing the spatial stratification of each site and the randomization of the 16 clusters and 160 plots.

The field data used in this study were collected using the Land Degradation Surveillance Framework (LDSF) [28,29,31] over the period 2005 to 2017 (Table 1). Data collection was conducted as part of a range of different projects coordinated by the authors. Observations of the presence or absence of visible erosion patterns (i.e., mass/gully, rill, and sheet erosion) from 171 one hundred km^2^ LDSF sentinel sites were included in the study, each site consisting of 16 one-km^2^ spatially stratified sampling clusters with 10 one-thousand-m^2^ plots per cluster (Figure 1). Within each plot (*N* = 26,091), four subplots with an area of 100 m^2^ were assessed for visible signs of erosion (see also Reference [32]). Erosion prevalence was scored at the plot level by summing up the number of subplots with visible signs of erosion, with 0 being no observed erosion and 4 being erosion observed in all four subplots. We took a cut-off at three subplots or more (>50%) to represent “severe” soil erosion and used this in the modeling of erosion prevalence. Vegetation structure was classified at the plot level as part of the LDSF surveys, using the Land Cover Classification System (LCCS) [33].

**Table 1:** Summary of the data included in the current study, showing the number of LDSF sites and countries by year.

Year	Number of LDSF Sites	Number of Countries
2005	2	2
2006	6	3
2007	6	1
2008	5	3
2009	6	4
2010	27	8
2011	26	9
2012	28	14
2013	23	12
2014	15	9
2015	17	12
2016	9	6
2017	14	8

### Processing of Remote Sensing Data

Remote sensing data from the Bidirectional Reflection Distribution Function (BRDF)-corrected MODIS (MCD43A4) product was applied in the current study, following Reference [29]. To obtain consistent annual composite reflectance values, we calculated the Soil Adjusted Total Vegetation Index (SATVI) [34] for each daily MCD43A4 image. Short-wave infrared (SWIR) bands have been found to be sensitive to both green and senescent vegetation [35], unlike the NDVI, which uses the near-infrared band instead of SWIR. Annual reflectance composites were created for each year between 2002 and 2017 based on the date of *SATVI_max_*in each MODIS pixel.

The pre-processing of the MODIS Earth observation data was conducted in Python, using the Google Earth Engine (GEE) [36] Python API. Processing steps included removal of clouds and water bodies (using the MODIS water mask product (MOD44W)). We also excluded areas with elevation values higher than 4300 m based on elevation data from the Shuttle Radar Topography Mission (SRTM) and excluded hyper-arid areas (deserts) from our analysis based on Tropical Rainfall Monitoring Mission (TRMM) [37] calibrated precipitation data for the period 1998 to 2014. Each annual MODIS composite was matched to the year that each LDSF site was surveyed (Table 2).

**Table 2:** Summary of LDSF plots (%) by vegetation structure class.

Vegetation Structure Class	LDSF Plots (%)
Cropland	34
Grassland	15
Woodland	12
Forest	10
Shrubland	10
Wooded grassland	8
Bushland	6
Other	4
Thicket	1

### Prediction Model for Mapping of Soil Erosion

Erosion prevalence was modeled in R Statistics [38] using a decision-tree approach known as Random Forests (RF) with 100 decision trees implemented as part of the *ranger* R library [39] to generate a classification model for soil erosion with MODIS reflectance bands 2 through 7 as independent variables in the model. In other words, only spectral data were used in the prediction model without other covariates meaning that a relatively low amount of input data is needed once the RF model has been trained. Model parameters were optimized or tuned using cross-validation as implemented in the *trainControl* function of the *caret* R library [40], followed by a tuning grid using the *expand.grid* function of the *caret* R library. The RF algorithm is a machine learning technique where multiple decision trees are constructed using bootstrap sampling of a training dataset and the trees in this ensemble are combined (bagged). This class of models was introduced by Reference [41] and has since been applied across a wide range of disciplines.

We used 70% of the LDSF plots to train the random forest (RF) model (*N* = 18,261). The remaining 30% of the LDSF plots (*N* = 7830) were used for model testing. Model performance was assessed by measuring the percentage of correctly classified test instances relative to observed instances, expressed as a confusion matrix [42]. We also calculated the Receiver Operating Characteristic curve (ROC) [43,44], which evaluates the accuracy of a model by considering errors that are either *false positives* or *false negatives*. A *true positive fraction* (TPF) is calculated as *TPF*(*c*) = *P*{*M* > *c*|*D* = 1}, where *M* is the probability of erosion in our case, which we defined as positive if it exceeded a fixed threshold *c*, while D was the binary outcome (erosion = yes/no). A *false positive fraction* (FPF) is then the probability that we falsely predict the occurrence of erosion where there is none. This is calculated as *FPF*(*c*) = *P*{*M* > *c*|*D* = 0}. The ROC curve is a graphical representation of the TPF against the FPF for all possible values of *c* (0 to 1). After assessing the various accuracy metrics of the RF model, we applied it to annual composite MODIS imagery to predict probabilities of soil erosion for the global tropics for the years 2002, 2007, 2012, and 2017, respectively.

### Assessment of Soil Erosion by Vegetation Cover, Holdridge Life Zone, Continent, and Sub-Continent

In order to better understand the variations in predicted erosion prevalence we randomly sampled the erosion prevalence map for 2017 using 1 million randomly generated points. We extracted the predicted prevalence of erosion for 2017 for each random point, along with the Holdridge life zone [45], continent, and sub-continent (as defined by the United Nations Statistics Division). The extracted values were then stored in a database and used to assess erosion across Holdridge life zones, continents, and sub-continents.

The Holdridge life zone system has a spatial resolution of one-half degree latitude and longitude and consists of 38 classes. The rationale for including this in our analysis of predicted soil erosion prevalence was that the system has been shown to reflect tropical vegetation zones well through the inclusion of annual precipitation (mm), biotemperature (humidity provinces), and potential evapotranspiration (PET) ratio as barycentric subdivisions. Hence, assessing soil erosion prevalence across these zones is useful in understanding bioclimatic factors that may drive soil erosion in tropical ecosystems.

## Results

**Figure 2:**
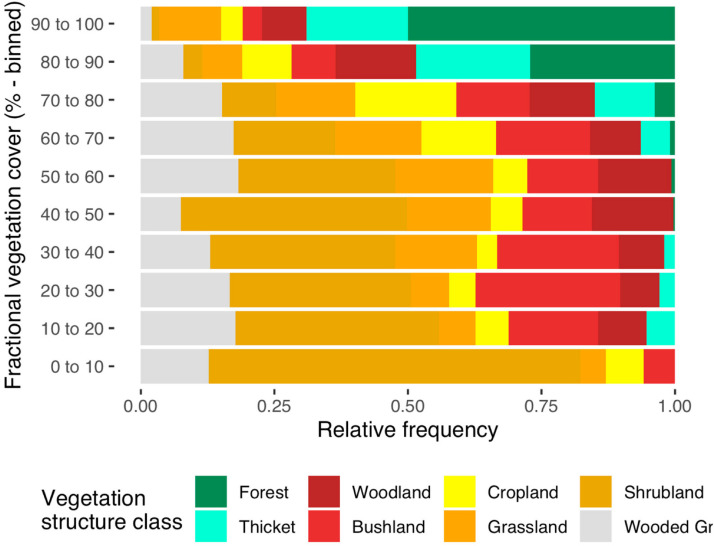
The relative frequency of maximum annual fractional vegetation cover (i.e., SATVImax) vegetation structure class in the dataset. The values were divided into 10 groups.

The LDSF data included in this study represented a range of different climate zones and ecosystems, as illustrated by the distribution of LDSF plots across various LCCS vegetation structure classes shown in Table 2 and Figure 2. The most dominant vegetation structure class was cropland (34%), followed by grassland (15%), woodland (12%), and forest and shrubland (10% each; Table 2).

As far as land cover is concerned, forest ecosystems had the highest fractional vegetation cover (i.e., *SATVI_max_*) on average as expected. Woodlands, shrublands, croplands, and grasslands showed a wide range of*SATVI_max_*values due to the high degree of mixture between senescent and green vegetation, as well as bare plots. Observed erosion prevalence also ranged widely for the sites included in our study, with a relative frequency of severe erosion of about 46% on average for the whole dataset.

### Prediction Model Performance

**Figure 3:**
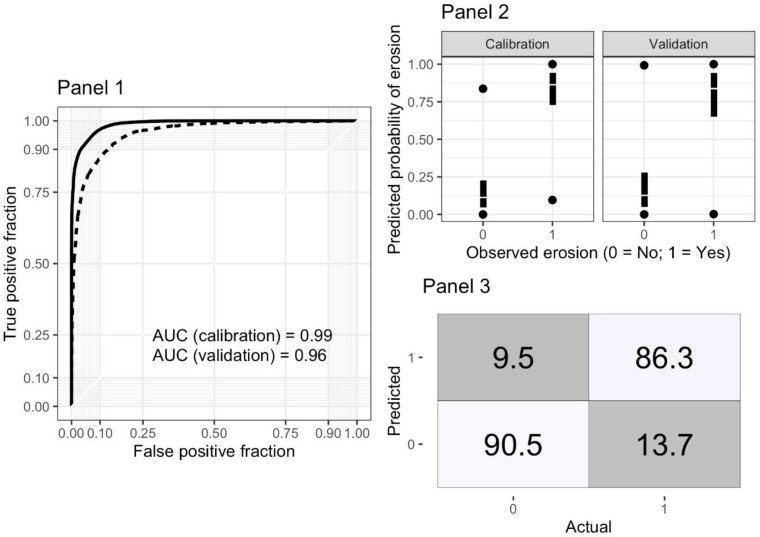
Model performance metrics for the RF prediction model, based on both calibration (training) and validation (testing) datasets. Panel 1 shows receiver–operator characteristics (ROCs), Panel 2 shows predicted probabilities versus observed erosion (0/1) as simple boxplots for both models’ runs, and Panel 3 shows a confusion matrix for predicted and observed (actual) erosion classes using the test dataset

Parameter tuning for the RF model showed optimal *mtry* and *nodesize* values of 1.5 and 5, respectively. Calibration model performance for mapping of erosion based on MODIS was good, with an out-of-bag (OOB) prediction error of 23.8% and an overall accuracy of the calibration model of over 90%. As shown in Figure 3 (Panel 1), the area under the ROC curve (AUC) was 0.99 for the calibration dataset and 0.96 when we tested this model on the 30% of LDSF plots that were held out for model validation or testing. Both the calibration and validation models were able to separate eroded and non-eroded plots well (Figure 3, Panel 2).

Model accuracy for the detection of erosion was about 86%, while accuracy for non-detection was somewhat higher at about 91%, on average (Figure 3, Panel 3). The model also showed good agreement with *K* >= 0.6, indicating good precision overall. These results show that we are able to predict the prevalence of soil erosion with good accuracy and prediction based on MODIS reflectance data. Calculations of variable importance in the calibration model, indicates that the red (3), NIR (4) and SWIR (6 and 7) bands are the most important variables for prediction of soil erosion from MODIS. The importance of these bands is likely due to the combination of their sensitivity to soil cover (NIR and red in particular), and senescent vegetation and bare ground (SWIR bands and NIR) [46].

Model performance was comparable to that reported by Reference [28] when applying similar modeling approaches to the local mapping of soil erosion based on Landsat in a case study from Ethiopia.

### Mapping Erosion Prevalence in the Global Tropics

**Figure 4:**
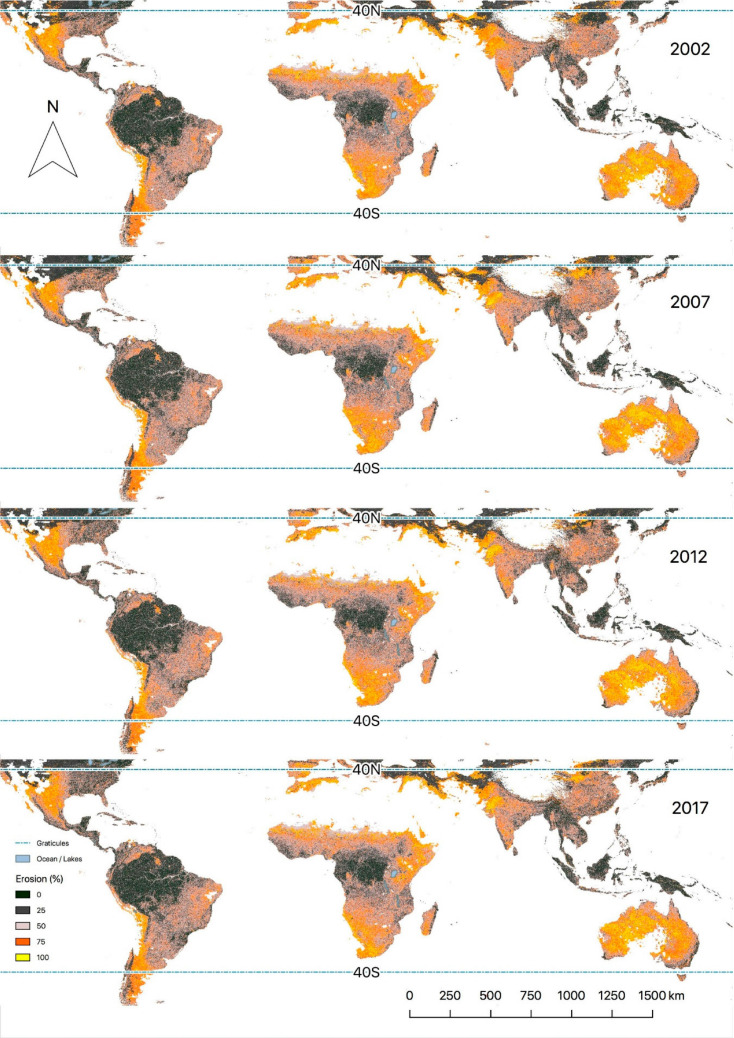
Predicted probabilities of soil erosion in the global tropics. The prediction model was developed based on MODIS imagery over the period 2005 to 2017, matching annual composites to the years of LDSF field data collection and fitted to annual composite reflectance data for 2002, 2007, 2012, and 2017. Hyper-arid (i.e., deserts) areas and areas with elevations higher than 4300 m were masked (i.e., white areas on the map).

Given the high levels of accuracy, we applied the RF model to the MODIS image library of annual reflectance composites for 2002, 2007, 2012, and 2017, producing spatial maps of predicted erosion prevalence at a 500 m resolution for each year covering the global tropics (Figure 4).

**Figure 5:**
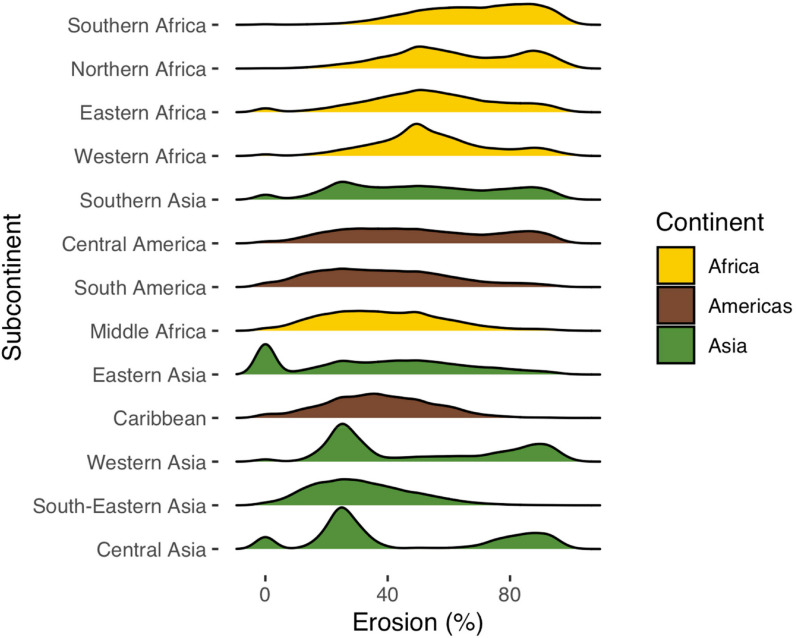
Predicted probabilities of soil erosion by continent and subcontinent (as defined by the United Nations Statistics Division) for 2017, showing the distribution of predicted erosion (%) based on 391,000 random points. The subcontinents were arranged according to median erosion prevalence. Southern Europe and Oceania were not included in the figure given the lack of validation data for these continents.

Our model estimates for 2017 showed a median of about 47% erosion when deserts were excluded. Erosion hotspots globally include semi-arid ecosystems in Kenya, Ethiopia, Somalia, southern Africa, western Africa, Australia, and Argentina, as well as fragile ecosystems in the Andes and the Tibetan plateau. Figure 5 shows a summary of predicted erosion prevalence rates by continent and subcontinent in the global tropics, based on a set of randomly sampled plots (*N* = 391,042 after excluding hyper-arid areas). We did not include Oceania and Europe in this analysis since we do not have observations of erosion on these continents, although the map in Figure 4 does extend to these continents. Median erosion prevalence was highest in southern Africa, followed by northern Africa, eastern Africa, and western Africa (Figure 5). However, as Figure 5 shows, there is a lot of variability in erosion prevalence within continents. Below we discuss some examples from contrasting parts of the tropics, including predictions for 2002 to 2017.

## Discussion

Earth observation offers opportunities for mapping and monitoring of soil erosion in landscapes that are unique in many ways. By utilizing information about the land surface captured across multiple parts of the electromagnetic spectrum, satellite data can be used to derive predictive models of often complex processes and patterns by using field observations to train these models. One such complex process is soil erosion, which can occur across a wide range of topographic, geomorphological, and land cover conditions. A key advantage of Earth observation in this regard is that we are able to derive accurate predictions of the severity and spatial extent of soil erosion using only information from the spectral bands of the sensor, which means that no additional covariates that may be time consuming to collect, expensive, or simply unavailable, are needed. This means that we can achieve results with unprecedented levels of consistency. The use of remote sensing in land surface process models not only provides useful information for numerical modelling but can also reduce the risk of propagating errors [47,48] from the derivation of covariate layers. An example where such errors may propagate is in the use of empirical models for predicting soil erosion such as the RUSLE which relies on factors or proxies derived from layers that may have large uncertainties associated with their estimation.

### Land Cover Effects on Soil Erosion

**Figure 6:**
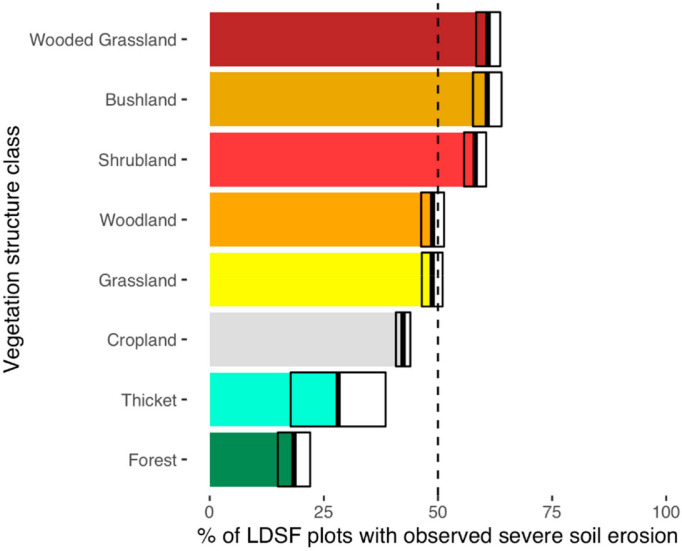
Relative frequency of observed erosions in LDSF field plots by vegetation structure class. The vegetation structure classes were arranged by frequency of observed erosions. Note that we did not include wetlands and restricted vegetation types. The crossbars show the mean +/- standard errors for the frequency of eroded plots in each vegetation structure class.

The role of vegetation cover in controlling rates of soil erosion by protecting soils from the impacts of rain drops (and wind) is widely accepted [16]. This is also reflected in the observations of erosion in this study, with the lowest observed erosion frequencies in forest ecosystems and thickets (Figure 6) where vegetation cover tends to be dense. Reference [49] reported similar results with lower rates of soil erosion under native forest vegetation than conventional agriculture based on compiled data from a range of different studies. We also observed high erosion rates in wooded grasslands, shrublands, and bushlands. Many of these ecosystems can have relatively high vegetation cover, but with high rates of observed erosion. Observed signs of erosion in croplands were somewhat lower than one might expect at about 42% of the surveyed plots. This could be the result of a number of factors such as plowing of fields, which may result in erosion patterns not being detected in cropland, although this was observed in a limited number of plots and was unlikely to have influenced much the prediction results. Also, the median slope for cropland plots was about 7% (4^◦^), which could be another factor resulting in an erosion being observed in a moderate number of plots.

**Figure 7:**
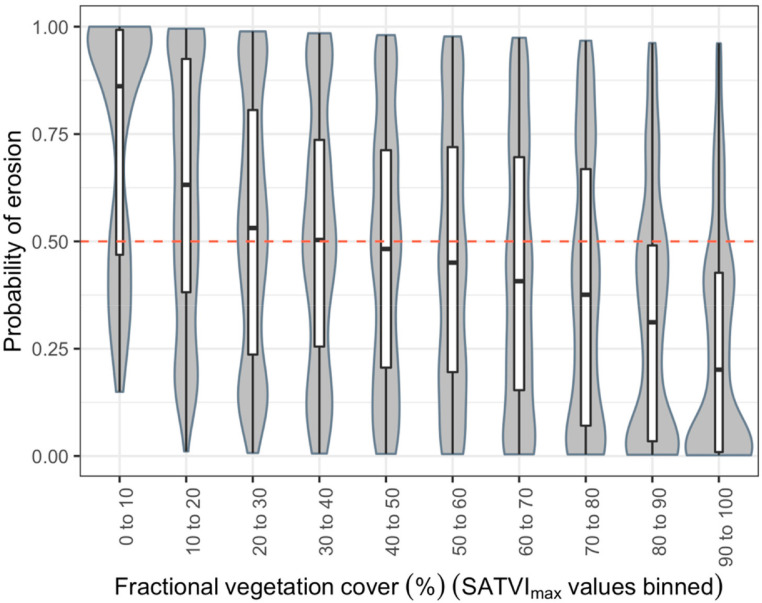
Violin plot with box-plot overlaid, showing the probability of observed erosions relative to fractional vegetation cover (SATVImax), divided into 10 categories. The probability of erosion is based on a generalized linear mixed-e_ects model with LDSF sentinel sites and clusters within sites as random e_ects.

To examine this further, we binned the *SATVI_max_*values into 10 categories and assessed these against the probability of erosion in each plot (Figure 7). As we see from Figure 7, the highest rates of erosion were observed where plots had *SATVI_max_*values less than 10%, as expected, while there was a high level of variability at between 10% and 50% fractional vegetation cover. Similarly, for higher than 50% fractional cover, erosion prevalence rates decreased, but again with high levels of variability until it reached 70% or higher fractional cover (Figure 7), beyond which erosion rates dropped strongly. This confirms that erosion prevalence can be high even when vegetation cover is relatively dense, such as in areas where invasive species are inhibiting the growth of native grasses or herbs in the understory due to allelopathic effects [50], resulting in low herbaceous cover despite high woody cover. These effects are often exacerbated due to the ability of many invasive species to thrive in areas that are degraded, often outcompeting native species in terms of access to available soil nutrients and/or water [51]. A concrete example of ecosystems where this occurs include areas with high prevalence of invasive woody species such as *Prosopis juliflora* in the degraded drylands of East Africa.

Given the above, methods used to detect soil erosion from remote sensing need to be able to accurately predict its occurrence under varying levels of vegetation cover as we may observe *SATVI_max_*values higher than 50%, but with rates of erosion higher than 60%, for example. Another implication of these findings is that land cover alone is not an adequate indicator or soil erosion, or land degradation.

There appear to be thresholds in fractional vegetation cover beyond which erosion reduces drastically, which will have implications for ecological regime shifts that can lead to a loss in ecosystem resilience and determine “flipping point(s)” [52], where an ecosystem enters into an alternative stable state [53] (e.g., through increased soil erosion as a response to vegetation cover loss). The predictions (maps) presented here can help in determining flipping points both in terms of thresholds beyond which soil erosion severity may lead to accelerated land degradation and in terms of determining temporal trends in erosion prevalence. Such thresholds should be studied in more detail in future studies, particularly in terms of the more general patterns presented here as compared to local processes that lead to increased soil erosion. By combining these maps and temporal trends with information on changes in factors such as land cover or land use, one can also assess the drivers of increased soil erosion that may lead to ecosystems flipping. Determining such flipping points is of critical importance for management, particularly in the case of land degradation, as the new (degraded) state may also be highly resilient, making restoration of the system difficult [51,54]. Future studies will need to investigate these relationships further, particularly interactions between woody and herbaceous cover fractions.

### Assessing Changes in Soil Erosion Prevalence over Time

**Figure 8:**
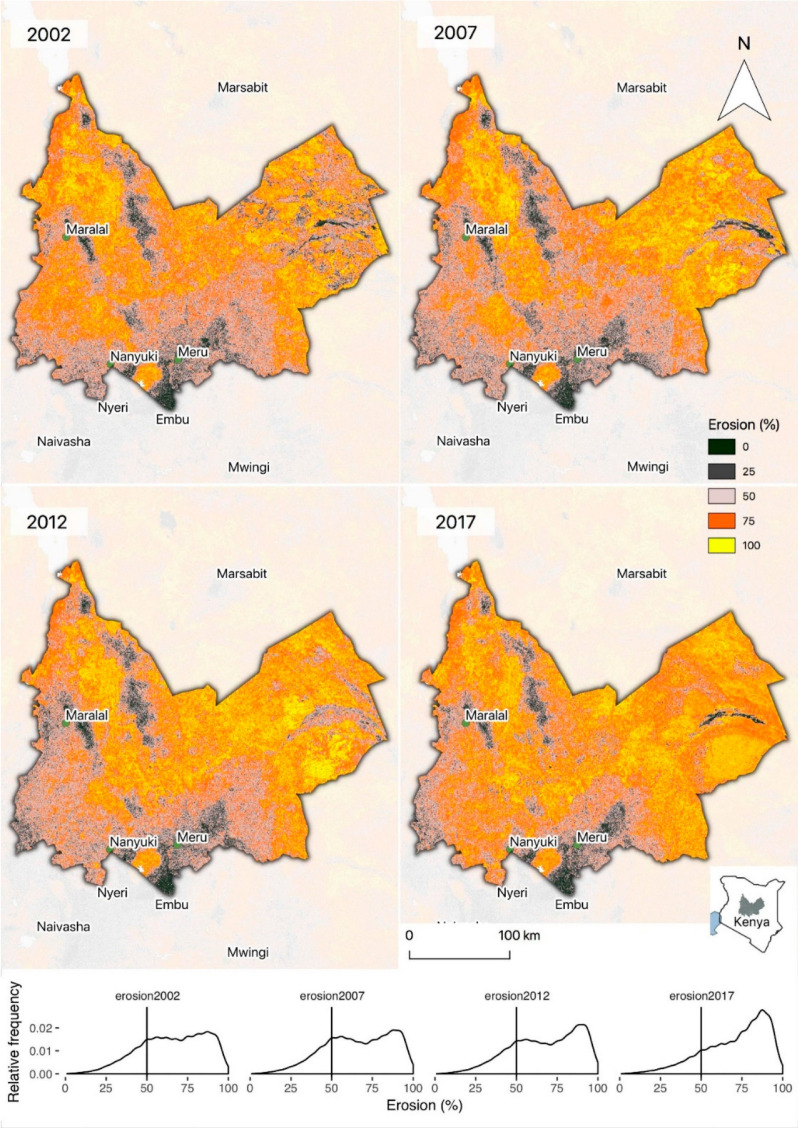
Predicted probabilities of soil erosion for Laikipia, Meru, Isiolo, and Samburu counties in central and northern Kenya. The region shown is outlined in gray relative to a map of Kenya below the legend on the right.

Closer examinations of the predictions presented for 2002, 2007, 2012, and 2017 for East Africa and India show varying patterns and trends. In East Africa 8, Laikipia, Meru, Isiolo, and Samburu counties of Kenya show moderate to high erosion prevalence, but with an increase in areas with high erosion prevalence (>75%, Figure 8, panel below maps) between 2002 and 2017, particularly in dry-lands. The majority of the areas shown in Figure 8 are rangeland systems. Other studies from eastern and southern Africa show that over-grazing and poor rangeland management have resulted in reduced grassland productivity [55,56], often leading to severe soil erosion and further declines in productivity in these ecosystems. Bush encroachment is a problem in some of the most severely eroded areas in Figure 8, exacerbating the situation further by leading to reduced herbaceous cover and increasing erosion. The drivers of soil erosion and land degradation in these systems are often complex, and may include increased population densities, demographic changes, and land tenure relations that restrict movement of pastoralists, and in some areas the introduction of unsustainable agricultural practices. Further, interactions with climate change have been reported in a number of studies, including more aggressive rainfall regimes that are likely to have important impacts on the rates of soil erosion [57–59].

**Figure 9:**
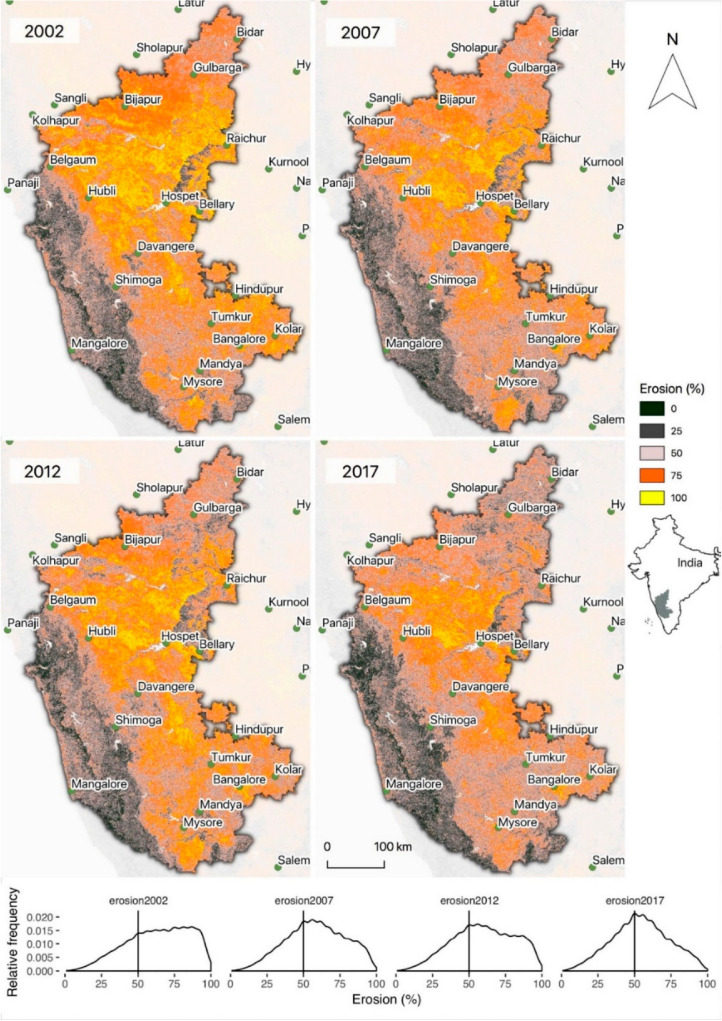
Predicted probabilities of soil erosion for Karnataka, India for 2002, 2007, 2012, and 2017. The location of Karnataka is shown outlined in gray on the map of India below the legend.

Figure 9 shows estimates of erosion prevalence for the Indian state of Karnataka. There appears to be less soil erosion in 2017 relative to 2002, in many parts of the state (see panel below maps in Figure 9). Assessments of actual trends will need to be made, but the findings presented here are consistent with studies conducted in this part of India, including Reference [60] which reported reductions in soil erosion in Karnataka, which were generally consistent with the estimates of our study (Figure 9). Assessments of soil erosion for the Kurnool watershed in 2006/2007 reported that 68% of this watershed was eroded, while in a more recent project report, extensive soil and water conservation, including agroforestry, were reported in Kushtagi taluk [61].

#### Erosion by Holdridge Life Zones

**Figure 10:**
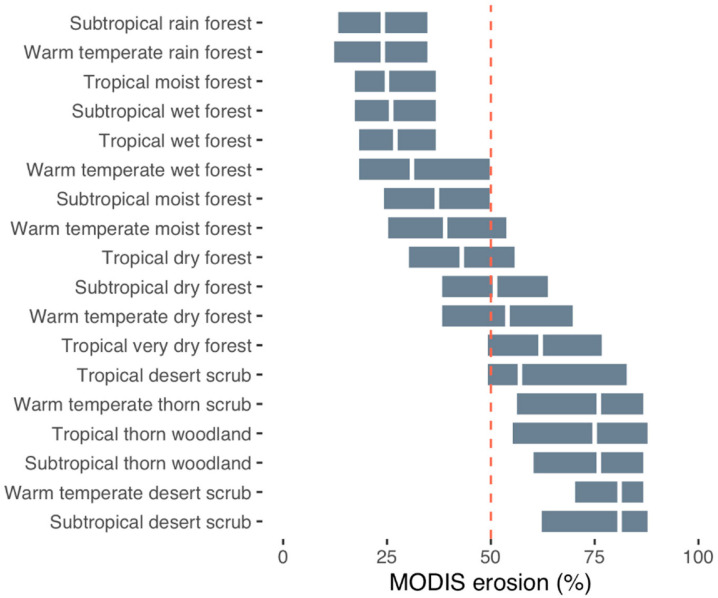
Predicted probabilities of soil erosion using Holdridge life zones in the tropics (excluding tropical and subtropical deserts), ranked from the lowest to highest median erosion in 2017.

Estimated rates of erosion by Holdridge life zones showed the highest predicted erosion prevalence values in desert scrub and “tropical thorn woodland” and “very dry forest” (Figure 10). The latter two life zones broadly correspond to what we label shrubland, (drier) wooded grassland, and grassland systems, and were also consistent with our field observations of erosion (Figure 6). These results point to the vulnerability of drier ecosystems in terms of land degradation [62] due to the presence of soil erosion because of high rainfall intensities, as discussed earlier for East Africa (Figure 8). If we consider the increasing human influence on these systems [63] due to the facts of population growth and agricultural expansion, coupled with climate change and increasingly erratic and, in many cases, rising levels of rainfall intensity [59], improved land management to reduce soil erosion is particularly important in these systems. Spatially explicit assessments of land degradation in dryland ecosystems are of critical importance for targeting interventions to restore degraded lands and monitor changes, given their vast spatial extent and the number of people that depend on them for their livelihoods. As expected, the lowest predicted erosion prevalence was found in moist and wet ecosystems, such as in rain forests (Figure 10).

The results of this study show that the Holdridge life zones provide a useful framework for assessing soil erosion risk since they integrate multiple bioclimatic factors, and these can be applied in analyses to better understand both risk as well as constraint envelopes for restoration of degraded ecosystems. Such assessments will be particularly important in the context of future climate scenarios and their potential influence on the different bioclimatic factors.

#### Limitations of the Presented Approach

While we have shown that the approach to mapping soil erosion in the current study can produce results with high levels of accuracy, there are limitations to the use of Earth observation for assessing these types of processes. A key limitation in our opinion is in assessing drivers of these processes, which will require a combination of both social and ecological data that can be challenging to detect remotely. Another limitation lies in unpacking the often complex biophysical interactions that lead to systems becoming more prone to erosion and land degradation in general, including the adaptive capacity of ecosystems. Finally, the predictions presented here represent estimates at a moderate spatial resolution. This is highly useful for assessments at regional, country or global scales, but has limitations in terms of applications for the restoration of degraded land where information at more detailed spatial scales will be needed. The use of higher resolution Earth observation data such as Landsat and Sentinel 2 will need to be explored.

## Conclusions

Soil erosion continues to adversely affect millions of hectares of land in the global tropics, resulting in losses in productivity and increased food insecurity, decreased resilience of ecosystems, and increased vulnerability to climate change. Its spatial extent is generally not well understood and interventions to limit erosion have had limited success due to the lack of appropriately targeted interventions, hampering progress towards preventing further land degradation. We used systematic field surveys based on a spatially balanced sampling design, analysis of Earth observation data and ensemble modeling techniques to map soil erosion prevalence at high levels of accuracy across a range of tropical ecosystems. The high model accuracy warranted the use of these models for estimating erosion prevalence in the global tropics based on satellite imagery from the MODIS platform.

The median erosion prevalence in 2017 was estimated at 47% when desert ecosystems were excluded, with hot-spots particularly in semi-arid ecosystems. The highest occurrences of erosion overall were found in southern and northern Africa with 70% and 60% predicted erosion, respectively. Median erosion prevalence in Eastern Africa was estimated at 54%, while for Western Africa, including the Sahel, it was 51% and for southern Asia 50%. The lowest median estimated erosion prevalence by subcontinent was found in Central Asia (29%) and south-eastern Asia (30%). Our predictions showed reductions in erosion in parts of India over the period 2002 to 2017 that can potentially be attributed to extensive soil and water conservation activities. In contrast, dryland systems in Kenya showed an increase in the severity and extent of erosion over the same period. Erosion was found to be high even under relatively high fractional vegetation cover in some cases, which has important implications for methods to assess soil erosion and land degradation in general.

Our study has important implications for management of ecosystem health in landscapes, specifically for the quantification and spatial prediction of soil erosion prevalence and the targeting of interventions to control erosion and reverse land degradation. Soil erosion is currently not featured as part of the indicator sets included in major global initiatives to tackle land degradation, such as the Bonn Challenge and targets set by the Parties of the UN Convention to Combat Desertification (UNCCD) to achieve Land Degradation Neutrality (LDN). Given the accuracy of the results presented in this study for detecting and mapping soil erosion based on Earth observation, soil erosion should be included as a key indicator in these and other international conventions on land degradation and desertification.
